# Impact of reconstruction type on late gadolinium enhancement scar quantification at 3-Tesla: Magnitude vs. phase-sensitive inversion recovery

**DOI:** 10.1016/j.jocmr.2026.102727

**Published:** 2026-04-15

**Authors:** Aida Moafi, Simran Shergill, David Adlam, Nilesh J. Samani, Gerry P. McCann, J. Ranjit Arnold

**Affiliations:** a Department of Cardiovascular Sciences, University of Leicester, the National Institute for Health and Care Research Leicester Biomedical Research Centre and British Heart Foundation Centre of Research Excellence, Glenfield Hospital, Leicester, UK; b Centre for Digital Health and Precision Medicine, University of Leicester, Leicester, UK

**Keywords:** Cardiac magnetic resonance imaging, Myocardial infarction, Scar quantification, Ischemic cardiomyopathy, Infarct burden, Full-width at half-maximum

## Abstract

**Background:**

The identification of myocardial scar using late gadolinium enhancement (LGE) cardiovascular magnetic resonance (CMR) is well established, providing diagnostic and prognostic value in both ischemic and non-ischemic cardiomyopathies. Phase-sensitive inversion recovery (PSIR) provides more consistent image contrast compared with magnitude (MAG) reconstructions. However, the impact of reconstruction method on scar quantification remains uncertain.

**Methods:**

One-hundred and thirty-six patients from three prospectively enrolled cohorts with chronic myocardial infarction (>3 months post-infarction), representing distinct infarct phenotypes, underwent contrast-enhanced research CMR. LGE images were acquired using either a breath-hold, segmented spoiled gradient-echo or free-breathing, motion-corrected, balanced steady-state free precession sequence. Infarction was quantified on matched, short-axis MAG and PSIR reconstructions using the full-width at half-maximum method, with total infarct size expressed in grams (g) and as percentage of left ventricular mass. The primary analysis compared scar quantification between the two reconstruction methods.

**Results:**

Compared with MAG, PSIR demonstrated significantly higher median scar mass (13.7 g [6.0–25.7] vs. 8.9 g [4.4–16.6], p<0.001) and percentage of left ventricular mass (14.2% [7.5–21.9] vs. 10.6% [5.4–14.2], p<0.001). Inter-method agreement was moderate for both absolute and relative scar mass (intraclass correlation coefficients: 0.76 [95% CI: 0.33, 0.89] and 0.74 [95% CI: 0.20, 0.89], respectively), with a systematic, proportional difference on Bland–Altman analysis, with consistently higher scar mass by PSIR (mean bias: +5.01 ± 5.87 g; limits of agreement: −6.50 to +16.51 g and +4.54 ± 4.62%; limits of agreement: −4.51 to +13.60%).

**Conclusion:**

Quantification using PSIR yields higher infarct sizes compared with MAG LGE reconstructions. This emphasizes the importance of using a consistent reconstruction method when quantifying scar burden.

## Graphical abstract

**Figure:**
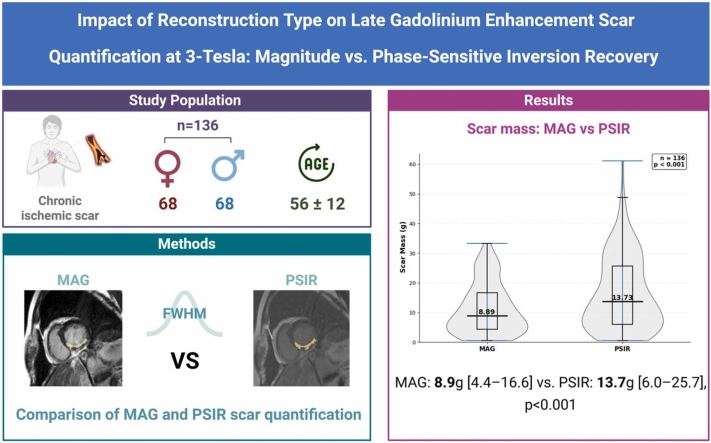

## 1 Introduction

Late gadolinium enhancement (LGE) imaging with cardiovascular magnetic resonance (CMR) is considered the non-invasive reference standard for identifying and quantifying myocardial scar across ischemic and non-ischemic cardiomyopathies. In chronic myocardial infarction, scar burden exceeding 5% of left ventricular (LV) mass confers a five-fold increased risk of death or ventricular arrhythmia [1], with scar extent and location providing independent prognostic value beyond established parameters such as ejection fraction [2]. Furthermore, scar burden underlies a number of post-infarct outcomes, including progression to heart failure and response to revascularization [3]. Accurate and reproducible quantification of scar burden is therefore essential to inform clinical decision-making, guide patient risk stratification and provide a robust, prognostically relevant endpoint in research studies.

Quantifying ischemic scar from LGE images typically relies on signal-intensity-based thresholding methods, the most common being the full-width at half-maximum (FWHM) technique, which defines scar as regions exceeding 50% of the maximum signal intensity within the infarct core [4]. The FWHM method is reproducible and compatible with commercially available software, yet its accuracy may be affected by the underlying image reconstruction technique. Conventional magnitude (MAG) inversion recovery sequences rely on selecting the appropriate inversion time to null normal myocardium. However, MAG imaging is prone to signal variation, with suboptimal nulling from incorrect inversion time selection increasing contrast variability, which may reduce the reliability to detect scar. Phase-sensitive inversion recovery (PSIR) reconstruction also assesses the polarity of magnetization [5], mitigating the need for accurate inversion time selection, resulting in more consistent image contrast and improved tissue differentiation. Therefore, differences in signal intensity scaling between MAG and PSIR reconstructions may affect quantitative scar assessment using threshold-based segmentation methods, resulting in variations in calculated scar size.

This study sought to compare MAG- and PSIR-derived scar mass in patients with chronic myocardial infarction to determine whether LGE reconstruction methods can be used interchangeably in clinical practice, and uniquely evaluates these differences across multiple sequences and cohorts with different infarct phenotypes, including the first analysis using a free-breathing, motion-corrected balanced steady-state free precession (MOCO-bSSFP) sequence.

## 2 Methods

### 2.1 Study population

One-hundred and thirty-six adult patients with chronic myocardial infarction who underwent research CMR in the chronic phase (>3 months post-infarction) were retrospectively identified from three prospectively enrolled cohorts at a single tertiary cardiac centre (Glenfield Hospital, Leicester, UK) with the inclusion/exclusion criteria as previously described (DREAM [Daily Remote Conditioning in Acute Myocardial Infarction; NCT0166461] [6], SCAD [Spontaneous Coronary Artery Dissection; UK National Registry] [7], and DISCORDANCE [Dobutamine vs. Adenosine CMR study; NCT03661827]). These cohorts represent distinct infarct phenotypes: DREAM (following ST-elevation myocardial infarction), SCAD (non-atherosclerotic dissection-related injury), and DISCORDANCE (non-ST-elevation infarction). All three studies received ethical approval from the UK National Research Ethics Service (DREAM, 12/EM/0304 [6]; SCAD, 14/EM/0056 [7]; and DISCORDANCE, 18/SC/0540). All participants gave written informed consent prior to study participation.

### 2.2 Cardiovascular magnetic resonance imaging

All CMR scans were performed at 3-Tesla (Skyra, Siemens Healthineers, Erlangen, Germany) with electrocardiographic gating. Cine imaging was acquired in the three long-axis views (4, 2, 3-chamber) and contiguous short-axis stack covering the ventricles from base to apex using a breath-hold, balanced steady-state free precession pulse sequence. LGE imaging was acquired 10–20 min after administration of 0.1 mmol/kg gadolinium-based contrast using either a breath-hold, segmented spoiled gradient-echo (GRE) (turbo-FLASH) [DREAM and SCAD cohorts] or free-breathing, motion-corrected balanced SSFP (MOCO-bSSFP) (TrueFISP) [DISCORDANCE cohort] sequence, with matched MAG and PSIR short-axis reconstructions. Contrast agents used were gadolinium-DTPA (Magnevist, Bayer Healthcare, Leverkusen, Germany) in the DREAM study; gadoterate meglumine (Dotarem, Guerbet, Villepinte, France) in the SCAD and DISCORDANCE studies.

### 2.3 Image analysis

CMR images were analyzed offline and blinded to participant details by a single observer (>3 years’ experience), using certified software (CVI42; Circle Cardiovascular Imaging, Calgary, Canada). For volumetric assessment, LV endocardial and epicardial contours were defined on the end-diastolic and end-systolic phases of the short-axis cine images to calculate end-diastolic volume, end-systolic volume, ejection fraction, and myocardial end-diastolic mass. For quantitative LGE assessment, LV endocardial and epicardial contours were manually drawn and applied to both reconstructions, using the same infarct core as the reference signal for thresholding. Exclusion zones were delineated to exclude non-infarct territories and minimize erroneous signal detection. Total infarct size was expressed in absolute (grams [g]) and relative terms (percentage of LV mass).

### 2.4 Statistical analysis

Continuous data are expressed as mean ± standard deviation for normally distributed variables or as median [Q1–Q3] if otherwise. Normality was assessed using the Shapiro-Wilk test. Categorical data are presented as counts and percentages (%). Paired sample t-test or Wilcoxon signed-rank tests were used to compare group differences, as appropriate. Correlation between scar size and ventricular parameters was assessed using Spearman’s correlation coefficient (r_s_). Agreement between methods was evaluated using intraclass correlation coefficients (ICC) with 95% confidence interval (CI) and Bland–Altman analysis. Statistical significance was defined as p<0.05. Statistical analyses were performed using Python 3.11 with SciPy 1.11 (Python Software Foundation, 2024).

## 3 Results

One-hundred and thirty-six patients (mean age 56 ± 12 years, 50.0% (68/136) female) with chronic myocardial infarction were studied (DREAM, n = 55, SCAD, n = 52, and DISCORDANCE, n = 29). Baseline characteristics for the entire cohort are presented in Table 1. Segmented GRE was performed in 79% (107/136) cases, whereas MOCO-bSSFP was performed in 21% (29/136) cases.

**Table 1:** Baseline and imaging characteristics

Variable	DREAM (n = 55)	SCAD (n = 52)	DISCORDANCE (n = 29)	Overall (n = 136)
*Demographics*				
Age, years	59.2±10.0	46.4±8.4	65.1±9.8	55.7±12.0
Female, n (%)	12/55 (21.8%)	52/52 (100.0%)	4/29 (13.8%)	68/136 (50.0%)
*Left ventricular function*				
Ejection fraction, %	56.8±6.4	55.5±6.9	35.3±12.0	51.7±11.7
End-diastolic volume, mL	191.0±42.4	153.5±28.1	217.7±71.3	182.4±51.9
End-systolic volume, mL	100.5±32.4	69.3±20.6	144.7±68.1	98.0±48.3
LV mass index, g	110.8±21.7	74.3±14.4	138.2±39.3	102.7±34.6
Scar burden*				
MAG (g)	13.4 [7.3–20.4]	5.3 [2.8–8.4]	13.5 [6.8–24.6]	8.9 [4.4–16.6]
PSIR (g)	21.7 [12.1–29.1]	6.5 [3.9–13.0]	16.8 [10.3–32.5]	13.7 [6.0–25.7]

Data presented as mean ± standard deviation, median [Q1–Q3] or counts (%)

*LV* left ventricular, *MAG* magnitude reconstruction, *PSIR* phase-sensitive inversion recovery

* p<0.001 for paired comparison of MAG vs. PSIR scar mass within each cohort

**Fig. 1:**
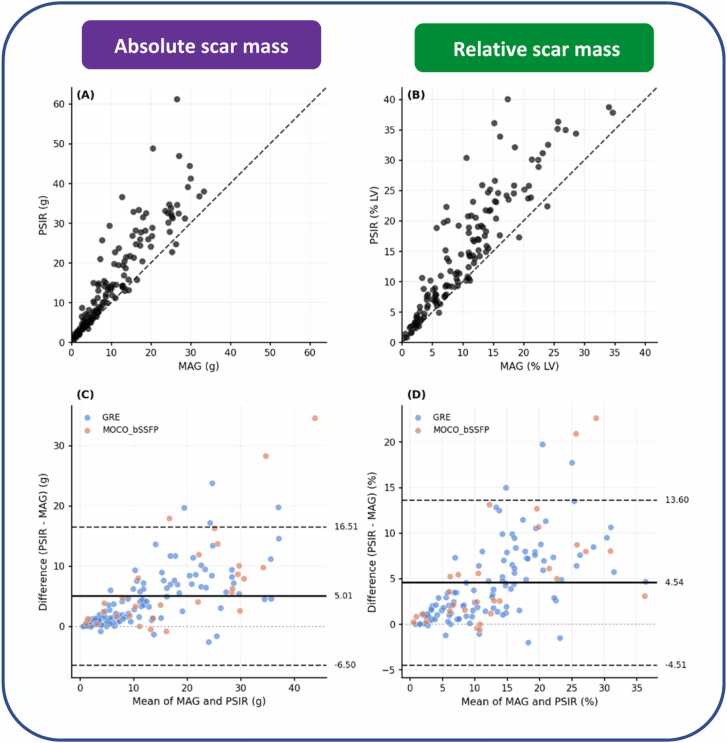
Comparison of MAG- and PSIR-derived scar quantification. (A, B) Scatter plots showing absolute scar mass in grams (A) and relative scar burden as percentage of LV mass (B). The dashed diagonal line indicates the line of identity (PSIR = MAG); points above the line reflect higher values on PSIR. (C, D) Bland–Altman plots showing the difference in scar quantification (PSIR − MAG) versus the mean of the two reconstructions for absolute scar mass (C) and relative scar mass (D). Mean bias was +5.01 g (limits of agreement: −6.50 to +16.51 g) for absolute scar mass and +4.54% (limits of agreement: −4.51 to +13.60%) for relative scar mass. *GRE* gradient-echo, *LV* left ventricular, *MAG* magnitude reconstruction, *MOCO-bSSFP* motion-corrected balanced steady-state free precession, *PSIR* phase-sensitive inversion recovery

In the overall cohort, median infarct size was significantly higher with PSIR compared with MAG for absolute scar mass (13.7 g [6.0–25.7] vs. 8.9 g [4.4–16.6], p<0.001) and relative to LV mass (14.2% [7.5–21.9] vs. 10.6% [5.4–14.2], p<0.001) **(**Fig. 1**A-B)**. For the primary analysis, the mean difference in scar (% of LV mass) was 4.5% [95% CI: 3.8–5.3], p<0.001. Cohort-level sensitivity analyses showed the same pattern, with PSIR consistently yielding higher scar mass than MAG (all p<0.001; Table 1).

Inter-method agreement between MAG and PSIR was moderate for both absolute (ICC 0.76 [95% CI: 0.33, 0.89]) and relative scar mass (ICC 0.74 [95% CI: 0.20, 0.89]), with Bland–Altman analysis demonstrating systematic, proportional difference, with consistently higher scar mass by PSIR (absolute mean bias: +5.01 ± 5.87 g, limits of agreement: −6.50 to +16.51 g and relative mean bias: +4.54 ± 4.62%, limits of agreement −4.51 to +13.60%) **(**Fig. 1**C-D**).

When analysis was stratified by sequence type, findings remained consistent**.** For segmented GRE, PSIR demonstrated significantly higher absolute (mean difference 4.42 g [95% CI: 3.50, 5.34]) and relative scar mass (mean difference 4.3% [95% CI: 3.5–5.1]) with moderate agreement (ICC 0.80 [95% CI: 0.33, 0.91] and ICC 0.76 [95% CI: 0.17, 0.90], respectively). For MOCO-bSSFP, PSIR also demonstrated significantly higher absolute (mean difference 7.19 g [95% CI: 4.00, 10.39]) and relative scar mass (mean difference 5.3% [95% CI: 3.1, 7.6]) with moderate agreement (ICC 0.66 [95% CI: 0.16, 0.86] and ICC 0.73 [95% CI: 0.21, 0.90]).

When assessing the associations between infarct size and LV functional indices, both MAG- and PSIR-derived scar mass correlated positively with end-diastolic (r_s_ = 0.62 and 0.59, both p<0.001) and end-systolic volume (r_s_ = 0.71 and 0.69, both p<0.001), and inversely with ejection fraction (r_s_ = −0.26 and −0.30, p = 0.002 and p<0.001, respectively).

## 4 Discussion

In this blinded study, we demonstrate that in patients with chronic myocardial infarction, PSIR consistently produces higher infarct sizes compared with conventional MAG reconstructions. This finding was consistent across traditional breath-hold segmented and newer, free-breathing sequences. To our knowledge, this represents the largest comparative evaluation of these sequences in patients with chronic infarcts. Unlike prior work, we provide a systematic assessment of inter-method differences across multiple cohorts with distinct infarct phenotypes, and are the first to assess these differences using the free-breathing MOCO sequence.

Comparing MAG and PSIR reconstructions, there was systematic, proportional difference in infarct burden, underscoring the importance of using a consistent reconstruction method for evaluating scar in clinical and research settings. Such systematic differences are particularly relevant when scar size is assessed longitudinally, as serial imaging with different reconstruction methods may exaggerate or mask true changes in scar burden. Furthermore, pooling data across studies using different reconstruction methods could lead to misinterpretation of treatment effects or associations with clinical outcome.

Previous studies comparing different thresholding methods, including FWHM and n-standard deviation, have reported similar reconstruction-dependent discrepancies. In a retrospective study of 80 patients with ischemic cardiomyopathy using a 1.5 Tesla system, scar metrics were significantly larger with PSIR compared with MAG across thresholding methods [8]. Another study of 40 patients with ischemic and 40 with non-ischemic cardiomyopathy showed that, using the FWHM method, MAG systematically underestimated scar volume compared with PSIR [9]. Unlike that study, which used single-shot LGE imaging, our work employed segmented and motion-corrected sequences, offering complementary evidence. In initial work by our group involving 40 patients following ST-elevation myocardial infarction [all of whom were included in the present analysis], we further demonstrated that these differences also exist in the acute setting (4–5 days post-infarct), with PSIR-derived median infarct size considerably higher than MAG (34.9 g [18.8–50.5] vs. 24.6 g [10.6–33.0], respectively, p<0.001) [10]. In the present analysis, we confirm that these differences between reconstruction methods persist in the chronic phase, and are consistent across commonly used clinical LGE sequences.

Despite differences in scar extent between MAG and PSIR reconstructions, both demonstrate a similar relationship with LV volumes and functional indices, which are well-established diagnostic and prognostic markers. However, the consistent, systematic differences in scar size may still influence clinical decision-making and reliability of findings between studies. This is particularly pertinent when scar burden is incorporated into radiomic and artificial intelligence-based analyses, as it may confound longitudinal assessments of infarct evolution.

**Fig. 2:**
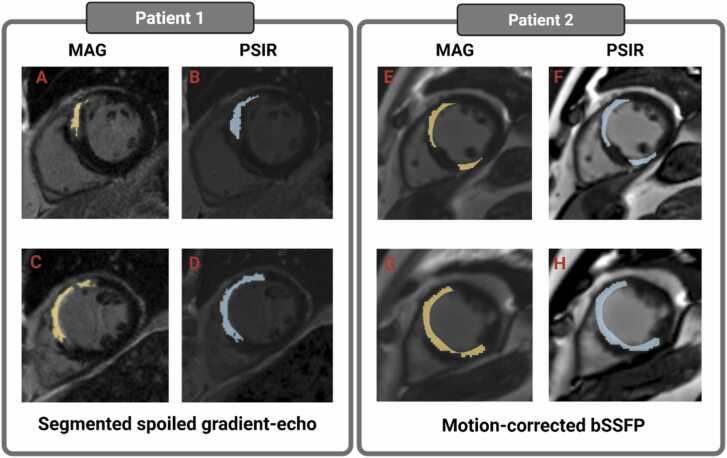
Representative short-axis LGE images with FWHM-derived scar segmentation comparing MAG (yellow) and PSIR (blue) reconstructions. Two slices from patient 1 imaged with spoiled gradient-echo (A–D) and two slices from patient 2 imaged with MOCO-bSSFP (E–H) are shown. MAG images demonstrate appropriate myocardial nulling. Despite acceptable image quality, PSIR yields visibly larger scar extent in both sequences, with patient-level PSIR − MAG differences of +17.17 g (spoiled gradient-echo) and +9.72 g (MOCO-bSSFP). *FWHM* full-width at half-maximum, *LGE* late gadolinium enhancement, *MAG* magnitude reconstruction, *MOCO-bSSFP* motion-corrected balanced steady-state free precession, *PSIR* phase-sensitive inversion recovery

Quantifying scar is dependent on the classification of hyperenhanced pixels, and thus differences in signal scaling between MAG and PSIR reconstructions directly affect the calculated scar size. From a technical standpoint, imperfect myocardial nulling in MAG reconstruction results in an additive signal offset in normal myocardium. This elevates the FWHM threshold calculated as the midpoint between maximum scar signal and minimum normal myocardial signal, leading to underestimation of scar extent. PSIR reconstruction preserves signal polarity and is therefore less sensitive to inversion time selection, which may explain the systematically larger scar measurements observed with PSIR in our study (Fig. 2). These intrinsic variabilities should be considered when conducting cross-sectional and longitudinal studies or when pooling data across reconstruction methods. Consistent use of a single reconstruction approach is essential, particularly for serial imaging studies or for radiomic and texture features that are sensitive to signal-intensity distribution.

## 5 Limitations

There are some key limitations in this study. First, while differences in scar size were observed across GRE and MOCO-bSSFP sequences, within-sequence differences could not be evaluated, as these were not acquired in the same patient. Second, test–retest reproducibility was not assessed, limiting our ability to evaluate the consistency of scar size by reconstruction method across repeated scans. All scans were performed at 3 Tesla using a single vendor platform; future studies across different field strengths and scanner systems would further strengthen the generalizability of these findings. Finally, without histological validation, it was not possible to determine which reconstruction most accurately reflects true infarct size. Future studies incorporating histological comparisons, where feasible, will be important to establish the most reliable method for quantifying myocardial scar.

## 6 Conclusions

PSIR yields systematically higher scar estimates than MAG reconstructions. Standardization of reconstruction method is essential to ensure reproducible quantification of scar, particularly when infarct size is used as a prognostic marker in clinical practice or when it serves as a research endpoint.

## Funding

AM is funded by a University of Leicester Future 100 Studentship. JRA was supported by a NIHR Clinician Scientist Award (CS-2018–18-ST2–007). GPM was supported by a NIHR Research Professorship (RP-2017–08-ST2–007).

## Author contributions

**Aida Moafi:** Writing – original draft, Visualization, Validation, Methodology, Investigation, Funding acquisition, Formal analysis, Data curation, Conceptualization. **Simran Shergill:** Writing – review & editing, Validation, Methodology, Data curation. **David Adlam:** Writing – review & editing, Resources, Funding acquisition. **Nilesh J. Samani:** Writing – review & editing, Resources, Funding acquisition. **Gerry P. McCann:** Writing – review & editing, Validation, Supervision, Resources, Funding acquisition, Conceptualization. **J. Ranjit Arnold:** Writing – review & editing, Validation, Supervision, Methodology, Investigation, Funding acquisition, Conceptualization.

## Declaration of competing interests

GPM is an editorial board member for JCMR, but was not involved in the peer review of this article and had no access to information regarding its peer review. Full responsibility for the editorial process for this article was delegated to another journal editor. The remaining authors have no competing interests to declare.
